# Preferential Selectivity of Inhibitors with Human Tau Protein Kinase Gsk3**β** Elucidates Their Potential Roles for Off-Target Alzheimer's Therapy

**DOI:** 10.1155/2013/809386

**Published:** 2013-10-10

**Authors:** Jagadeesh Kumar Dasappa, H. G. Nagendra

**Affiliations:** Department of Biotechnology, Sir M. Visvesvaraya Institute of Technology, Hunasamaranahalli, Via Yelahanka, Bangalore 562157, India

## Abstract

Alzheimer's disease (AD) is a neurodegenerative disorder characterized by the accumulation of amyloid beta peptides (A*β*) and neurofibrillary tangles (NFTs). The abnormal phosphorylation of tau leads to the formation of NFTs produced by the action of tau kinases, resulting in the loss of neurons and synapse, leading to dementia. Hence, tau kinases have become potential drug target candidates for small molecule inhibitors. With an aim to explore the identification of a common inhibitor, this investigation was undertaken towards analyzing all 10 tau kinases which are implicated in phosphorylation of AD. A set of 7 inhibitors with varied scaffolds were collected from the Protein Data Bank (PDB). The analysis, involving multiple sequence alignment, 3D structural alignment, catalytic active site overlap, and docking studies, has enabled elucidation of the pharmacophoric patterns for the class of 7 inhibitors. Our results divulge that tau protein kinases share a specific set of conserved structural elements for the binding of inhibitors and ATP, respectively. The scaffold of 3-aminopyrrolidine (inhibitor 6) exhibits high preferential affinity with GSK3*β*. Surprisingly, the PDB does not contain the structural details of GSK3*β* with this specific inhibitor. Thus, our investigations provide vital clues towards design of novel off-target drugs for Alzheimer's.

## 1. Introduction 

Alzheimer's disease is the most common form of neurodegenerative disorders [1] characterized by the formation of extracellular deposits composed of amyloid beta peptide (A*β*) [2] and masses of paired, helically wound protein filaments in the cytoplasm of neuronal cell bodies and neuritic processes called neurofibrillary tangles [3]. These NFTs are formed as a result of hyperphosphorylation of tau protein [4]. The tau proteins are phosphoproteins whose levels of phosphorylation are regulated by tau kinases and phosphatases [5]. Substantial evidence reveals the increased activity of glycogen synthase kinase 3*β* (GSK3*β*) (also known as human tau protein kinase I) during AD. Similarly, P25/cyclin-dependent kinase 5 (Cdk5), dual-specific tyrosine [Y] regulated kinase 1A (Dyrk1A), and mitogen-activated protein kinases (MAPK) also possess higher activity in AD brain [6]. 

 Thus, our work focuses on the chosen 10 kinases involved in hyperphosphorylation of tau and elevated responses in AD [7]. Kinase holds a large gene family and these domains are alike in sequence and structure. Developments of discriminating inhibitors are a key task in drug discovery and development, and appreciating the basis of kinase inhibitor selectivity is critical to the design of effective drugs. 

 GSK3*β* is composed of three domains: an N-terminal domain consisting of a closed *β*-barrel structure, a C-terminal domain containing a “kinase fold” structure, and a small extradomain subsequent to the C-terminal domain. The catalytic site is between the two major domains and has an ATP analogue molecule in its ATPbinding site. The adenine ring is buried in the hydrophobic pocket and interacts specifically with the main-chain atoms of the hinge loop [8]. The structure of GSK3*β* is known to have a catalytically active dimer conformation that progressively phosphorylates substrates with Ser/Thr penta repeats [9]. It is known that the inhibitors compete with the ATP binding sites of GSK3*β* [10, 11]. Realizing the need to design the inhibitors for these kinases, with the hope that it could affect the hyperphosphorylation of tau, the investigations have been undertaken. The studies reveal shared conservations across sequences, structural homologies, and ATP binding site geometries across the ten tau kinases. Interestingly, the inhibitor 3-aminopyrrolidine scaffold exhibits high preferential affinity with GSK3*β*. Though the literature on AD indicates overexpression and activity of GSK3*β* during pathogenesis of AD, surprisingly, the PDB does not contain the structural details of GSK3*β* with these specific inhibitors. Consequently, our explorations provide vital clues towards design of novel off-target drugs for AD.

## 2. Materials and Methods 

**Figure 1:**
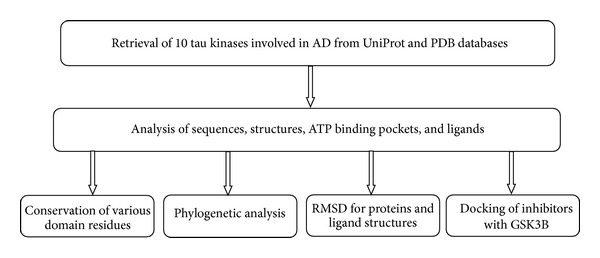
Work flow chart.

**Figure 2:**
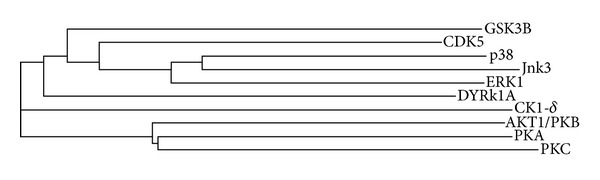
Phylogenetic tree generated for sequences of the tau kinases using CLUSTALW software.

The analysis of the structures of the 10 tau kinases, along with their respective ligands, was carried out as illustrated in the flow chart (Figure 1). To begin with, the structures of the 10 tau kinases, along with their respective ligands, were retrieved from the PDB and their details are provided in Table 1. A Phylogenetic tree was generated for the amino acid sequences of 10 tau kinases using CLUSTALW [12] and is illustrated in Figure 2. The dendrogram reveals that GSK3*β*, CDK5, MAPK such as p38, extracellular signal-regulated kinases 1 & 2 (ERK 1/2), c-Jun N-terminal kinase (JNK), and dual-specificity tyrosine-[Y]-phosphorylation-regulated kinase 1A (DYRK1A) are closely related (as in cluster 1); protein kinase A (PKA), protein kinase B (AKT/PKB), and protein kinase C (PKC) form the closely related second cluster. Interestingly, casein kinase 1 delta (CK1d) stands out as an independent taxis, indicating characteristic sequence variations from the group. 

**Figure 3:**
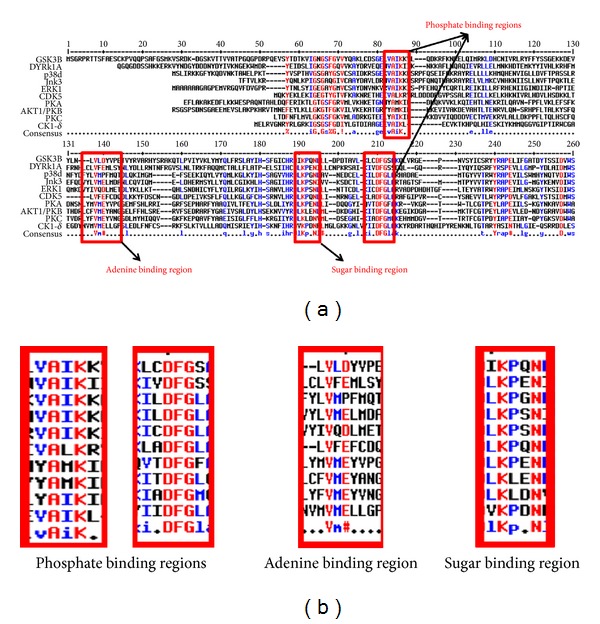
(a) Alignment of tau kinases showing the conserved residues in the ATP binding region. (b) Highlight of sequence conservation across ATP binding residues.

To observe the conservation of residues in the ATP binding region, a multiple sequence alignment was carried out amongst the 10 kinases, using the tool MultAlin [13]. The alignment is depicted in Figure 3(a), which reveals that the sequences differ significantly at the positions of gate-keeper residues and the surface residues, while they are well conserved at the subsites that interact directly with ATP. The ATP binding amino acids LYS 85 & ASP 200, ASP 133 & VAL 135 and GLU 185 are conserved across these kinases, and lie in the phosphate binding region, adenine binding region and in the sugar binding regions respectively (refer to Figure 3(b)). Overall sequence identity and similarity amongst the sequences and RMSD between the 3D geometries are highlighted in Table 2. The identity (and similarity) amongst the kinase sequences lies in the broad range of 22.8% (48.1%) and 49% (77.4%). The closest set appears between ERK1/2 and P38, which share the highest identity of 49%, while the least values of 22.8% exist between 2 sets, namely, CK1d and CDK5 and AKT and ERK1/2, respectively. The structural alignment to calculate the RMSD values was carried out using MultiProt [14]. It is interesting to note that the RMSD between all pairs of 10 tau kinases for C*α* atoms range between 1.07 and 1.82 Å (refer to Table 2). Interestingly, these lowest and highest values of RMSD are related to the molecule PKC. However as expected, for the molecule CK1d which stands out in the phylogeny, the identities with all other kinases are less than 30%. Similarly, the RMSD values of all the kinases with CK1d lie in the higher range of 1.57 to 1.76 Å. Further, the structural alignments revealed that the active site occurs between two lobes of kinases showing the conserved overlap of the ATP binding regions. Thus, the RMSD values for the entire ATP binding region amongst the kinases lie between 0.63 and 1.25 Å. However, the RMSD values for the phosphate binding region lie between 0.23 and 0.46 Å, and the nucleotide binding region is in-between 0.16 and 1.25 Å respectively. Hence, docking the binding site of kinases with ATP was attempted to appreciate the nature of interactions.  Table 3 provides the molecular details and properties of the various inhibitors used in the study. Their relative binding affinities in terms of IC50 and *K*_*i*_ values are also indicated for each inhibitor (Table 3). The set of chosen inhibitors does contain two small molecules which are deposited in the drug bank, that is, inhibitor 2 (Drug Bank ID DB07794) and inhibitor 5 (Drug Bank ID DB07919). In order to appreciate the structural similarity of these inhibitors with ATP, 3D alignments were carried out across the 7 kinase inhibitors and ATP. The outcome of these structural comparisons revealed that the RMSD between ATP and the 7 ligands lie between 0.37 and 0.67 Å, while the inhibitor-to-inhibitor RMSD values are in the range of 0.32 to 0.67 Å. All the RMSD values are consolidated in Table 4. 

The ATP binding site of GSK3*β* spans a long stretch containing 22 residues.  Table 5 lists the various residues forming the ATP binding pocket and exhibiting favorable interactions with ATP. In order to appreciate the conservation of this ATP binding site across all 10 tau kinases, ATP molecule was docked and all plausible interactions within 6 Å were tabulated. Docking studies were carried out using the Discovery Studio software Version 3.5 (Accelrys Software Inc., USA) and Lead IT tool of FlexX 2.1.2. It is clear from Table 5 that G63, A83, K85, E97, D/E133, N186, and D200 form the set of well-conserved residues in the ATP binding pocket and interact with ATP in all the 10 kinases. Though the molecules JNK and ERK1/2 exhibit less number of interactions with ATP, key contacts of conserved residues are indeed present. Residue corresponding to V110 exhibits least number of interactions across kinases. Though the residue corresponding to R141 appears changed in all the kinases, its interactions with ATP, across the receptors, are well conserved. 

Our aim to study the efficacies of select inhibitors with GSK3*β* triggered the need to dock these ligands to the key tau kinase GSK3*β*. Identifying the ATP binding site, the molecular docking studies of kinase inhibitors were carried out using the Discovery Studio software Version 3.5 and Lead IT tool of FlexX 2.1.2. GSK3*β* receptor structure was docked to all of the seven ligands to the ATP binding pocket by the rigid receptor-flexible ligand docking competencies of FlexX and the interactions are tabulated in Table 6. The docking examination revealed that the inhibitor-3-aminopyrrolidine scaffold exhibits high preferential affinity with GSK3*β*. While all inhibitors appear to sit in the ATP pocket of GSK3*β*, the most favorable is inhibitor 6 and the least probable is inhibitor 5. 

## 3. Results and Discussion 

In the present study, the analysis of 10 tau kinases implicated in AD has been performed to elucidate the conservation of the binding site and selectivity of the 7 inhibitors. Multiple sequence alignment, 3D structural alignment, catalytic active site overlap, and docking studies of inhibitors with GSK3*β* have been carried out to fingerprint the interactions with the key/gatekeeper residues in the ATPbinding pocket. The results highlight that tau protein kinases share common structural elements for the binding of the inhibitors and ATP. Comparatively, the inhibitor 3-aminopyrrolidine (inhibitor 6) exhibits high preferential affinity with GSK3*β*. Interestingly, the literature on AD indicates the overexpression and activity of GSK3*β* during pathogenesis of AD, and surprisingly, the PDB does not contain the structural details of GSK3*β* with this specific inhibitor. Our studies disclose that regions of the active site which found high conservation across tau kinases may form the determinants for binding to the ligand. Interactions of 7 inhibitors with the remaining 9 tau kinases in AD are also being explored.

## 4. Conclusions

 Our results indicate that the binding pocket of the 10 tau kinases is structurally conserved and offer a common feature of determinants with the 7 inhibitors investigated. Specific analysis with GSK3*β* reveals preferential binding to 3-aminopyrrolidine scaffold. This study highlights that suitable therapeutics can be successfully developed from the available chemical space, thus facilitating inhibitor design and off-target effects for AD. 

## Figures and Tables

**Table 1:** Details of 10 tau kinases used in the analysis.

Sl. number	Protein name	UniProt ID	PDB ID	Number of residues	Ligand present in the PDB structure	Nomenclature of inhibitors in the study
(1)	Glycogen synthase kinase 3 beta (GSK3*β*)	P49841	1J1C	420	Adenosine-5′-diphosphate	—
(2)	Cyclin-dependent kinase 5 (CDK5)	Q00535	3O0G	292	4-Amino-2-[[4-chlorophenyl]amino]-1,3-thiazol-5-yl][3-nitrophenyl]methanone	Inhibitor 1
(3)	p38 delta kinase (p38)	O15264	3COI	365	No Ligand	—
(4)	Mitogen-activated protein kinase 1 (Erk1/2)	P28482	1TVO	360	5-[2-Phenylpyrazolo[1,5-a]pyridin-3-yl]-1h-pyrazolo[3,4-c]pyridazin-3-amine [Drug Bank ID DB07794]	Inhibitor 2
(5)	Mitogen-activated protein kinase 10 (JNK3)	P53779	2O0U	464	N-{3-Cyano-6-[3-[1-piperidinyl]propanoyl]-4,5,6,7-tetrahydrothieno[2,3-c]pyridin-2-yl}1-naphthalenecarboxamide	Inhibitor 3
(6)	Casein kinase 1 delta (CK1d)	P48730	3UYT	415	4-[1-Cyclohexyl-4-[4-fluorophenyl]-1H-imidazol-5-yl]pyrimidin-2-amine	Inhibitor 4
(7)	Dual-specificity tyrosine-[Y]-phosphorylation-regulated kinase 1A (DYRK1A)	Q13627	3ANR	763	7-Methoxy-1-methyl-9h-beta-carboline [harmine complex] [Drug Bank ID DB07919]	Inhibitor 5
(8)	Protein kinase A (PKA)	P17612	3MVJ	351	[3R]-1-[5-Methyl-7H-pyrrolo[2,3-d]pyrimidin-4-yl]pyrrolidin-3-amine	Inhibitor-6 (3-aminopyrrolidine scaffold)
(9)	Protein kinase B (PKB/AKT)	P31749	3MV5	480	[3R]-1-[5-Methyl-7H-pyrrolo[2,3-d]pyrimidin-4-yl]pyrrolidin-3-amine	Inhibitor-6 (3-aminopyrrolidine scaffold)
(10)	Protein kinase C alpha (PKC)	P17252	3IW4	672	3-[1H-Indol-3-yl]-4-[2-[4-methylpiperazin-1-yl]quinazolin-4-yl]-1H-pyrrole-2,5-dione	Inhibitor 7

**Table 2:** Percentage identity, similarity, and RMSD values (for the overlapping number of atoms).

	GSK3*β*	CDK5	P38	ERK1/2	JNK	CK1d	DYRK1A	AKT	PKA
CDK5	^ #^35.6% ^@^66.0% *1.25 (238)	—	—	—	—	—	—	—	—
P38	32.3% 62.0% *1.45 (221)	36.4% 64.9% *1.52 (202)	—	—	—	—	—	—	—
ERK1/2	32.8% 64.5% *1.43 (252)	37.6% 68.0% *1.42 (233)	49.0% 77.4% *1.60 (277)	—	—	—	—	—	—
JNK	28.0% 54.8% *1.59 (238)	35.4% 64.3% *1.61 (228)	47.7% 75.3% *1.65 (265)	43.1% 73.8% *1.46 (287)	—	—	—	—	—
CK1d	25.3% 49.6% *1.64 (205)	22.8% 48.1% *1.60 (200)	25.6% 54.0% *1.72 (167)	29.5% 50.4% *1.75 (195)	24.6% 50.8% *1.76 (188)	—	—	—	—
DYRK1A	28.4% 53.2% *1.29 (253)	30.4% 7.5% *1.34 (243)	29.1% 53.6% *1.52 (218)	28.3% 57.1% *1.39 (246)	28.9% 52.3% *1.72 (247)	24.5% 51.8% *1.57 (213)	—	—	—
AKT	27.0% 48.4% *1.55 (222)	25.4% 57.4% *1.47 (214)	26.1% 56.7% *1.61 (205)	22.8% 53.1% *1.48 (231)	26.3% 52.5% *1.64 (221)	25.8% 54.5% *1.68 (207)	26.0% 56.4% *1.36 (230)	—	—
PKA	26.4% 59.2% *1.56 (213)	23.4% 58.9% *1.50 (207)	26.6% 59.4% *1.65 (183)	24.1% 53.9% *1.65 (214)	26.3% 52.9% *1.72 (194)	26.1% 53.8% *1.76 (207)	26.0% 57.1% *1.50 (219)	45.6% 77.2% *1.21 (292)	—
PKC	24.8% 51.8% *1.52 (223)	27.8% 61.9% *1.45 (214)	27.9% 56.4% *1.65 (203)	23.9% 56.2% *1.68 (228)	24.6% 50.8% *1.82 (221)	23.5% 55.8% *1.70 (208)	25.9% 56.7% *1.43 (224)	47.4% 79.2% *1.07 (285)	32.8% 60.3% *1.37 (279)

^#^Identity, ^@^similarity, and *RMSD (total number of atoms involved).

**Table 3:** Structures of 7 potential small molecule inhibitors and their relative binding affinities (IC_50_ and *K*_*i*_) values.

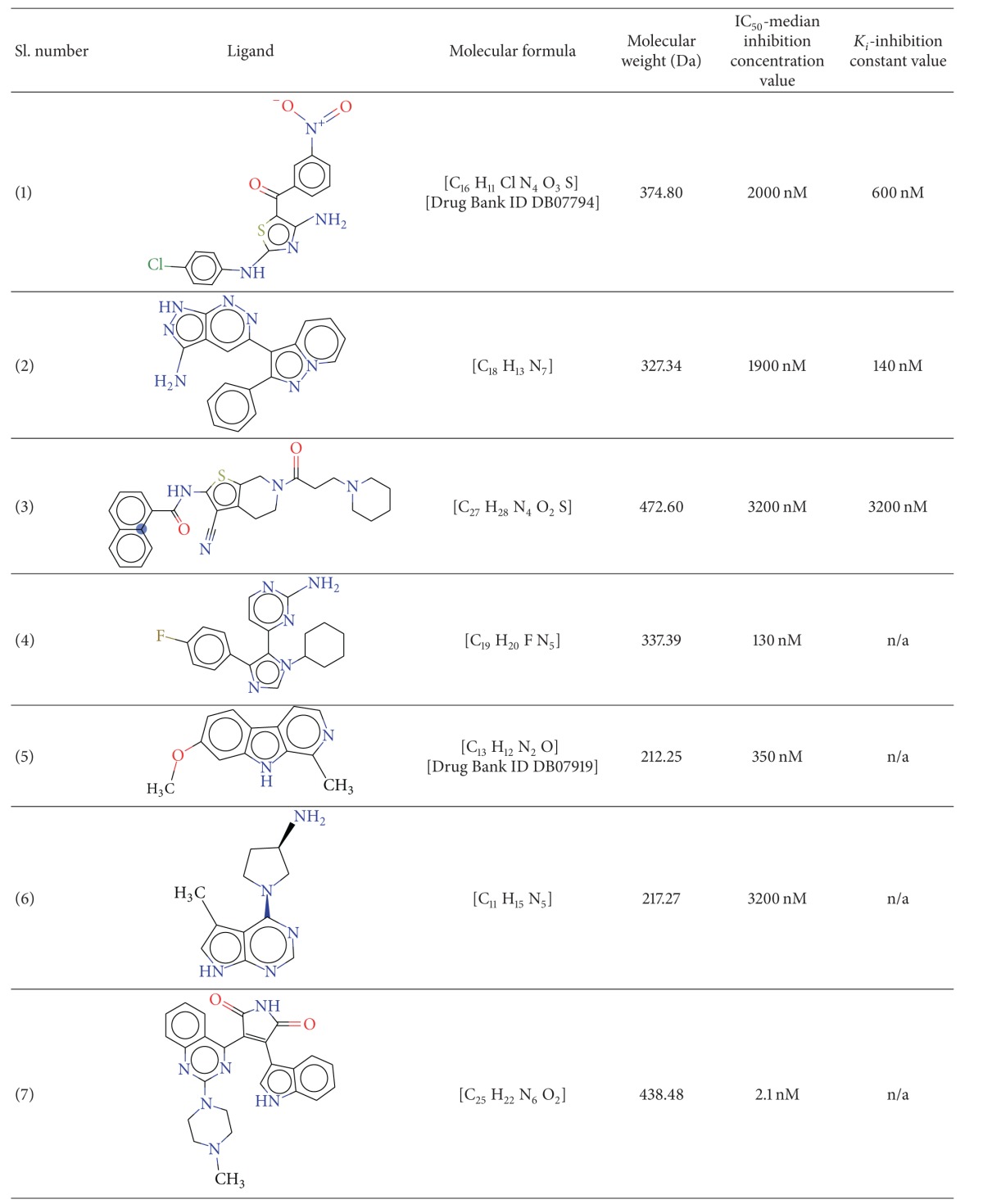

**Table 4:** RMSD values of ATP and the seven kinase inhibitors amongst each other.

	Inhibitor 1	Inhibitor 2	Inhibitor 3	Inhibitor 4	Inhibitor 5	Inhibitor 6	Inhibitor 7
Inhibitor 1	—	—	—	—	—	—	—
Inhibitor 2	0.57	—	—	—	—	—	—
Inhibitor 3	0.46	0.32	—	—	—	—	—
Inhibitor 4	0.56	0.66	0.54	—	—	—	—
Inhibitor 5	0.43	0.41	0.33	0.52	—	—	—
Inhibitor 6	0.51	0.39	0.46	0.49	0.49	—	—
Inhibitor 7	0.53	0.54	0.41	0.54	0.41	0.55	—
ATP	0.37	0.64	0.58	0.54	0.51	0.67	0.48

**Table 5:** Interaction of binding site residues amongst the 10 tau kinases with ATP.

GSK3*β*	CDK5	P38	ERK1/2	JNK	CK1d	DYRK1A	AKT	PKA	PKC	ATP binding site conservation across the 10 kinases
^ #^I 62 *[4.4]	I10 [5.70]	V31 [4.24]	I 31 [5.59]	I 70 [4.23]	I 15-[2.36]	I 165-[3.09]	L156 [4.34]	L49 [4.10]	L345 [4.70]	I/V/L
^ #^G 63 *[5.4]	G11 [4.7]	G32 [3.97]	G32 [>6]	G71 [4.31]	G16 [3.24]	G166 [5.12]	G157 [3.95]	G50 [4.88]	G346 [4.81]	G-well conserved
^ #^N 64 *[5.24]	—	S33 [3.93]	E33 [>6]	—	—	K167 [4.60]	K158-[2.95]	T51 [2.76]	K347 [5.88]	Not conserved
^ #^G 65 *[4.03]	—	G34 [3.36]	Y36 [2.79]	—	—	G168 [5.72]	G159 [5.08]	G52 [3.90]	G348 [>6]	Not conserved
^ #^S 66 *[2.82]	—	A35 [3.64]	—	—	—	S169 [>6]	—	S53 [4.59]	S349 [5.81]	Not conserved
^ #^F 67 *[3.21]	—	Y36 [3.17]	—	—	F20 [5.54]	F170 [>6]	F161 [5.54]	F54 [3.82]	—	Not conserved
^ #^V 70 *[5.9]	V18-[2.72]	V39 [2.89]	V39 [2.79]	V78 [>6]	I 23 [5.07]	V173 [>6]	V164 [4.93]	V57 [2.88]	V353 [4.80]	V/I
^ #^A 83 *[5.46]	A31 [4.61]	A52 [4.93]	A52 [4.64]	A91 [5.99]	A 36 [5.42]	A186 [5.00]	A177 [4.90]	A70 [4.49]	A366 [4.51]	A-well conserved
^ #^K 85 *[2.64]	K33 [4.28]	K54-[2.67]	K54-[2.93]	K93 [5.18]	K38 [3.23]	K188 [3.19]	I179 [5.23]	K72-[3.72]	K368-[2.31]	K-well conserved
^ #^E 97 *[4.59]	E51 [>6]	E72 [4.96]	E71 [3.71]	E111 [5.66]	E52 [3.78]	E203 [3.24]	E198 [4.93]	E91 [5.32]	E387 [3.45]	E-well conserved
^ #^V 110 *[5.1]	V64 [>6]	I85 [>6]	I84 [>6]	I124 [>6]	P66 [>6]	V222 [5.80]	T211 [4.13]	V104 [5.40]	T401 [3.29]	V/I/P/T
^ #^L 132 *[5.7]	F80 [4.31]	M107 [5.06]	Q105-[2.29]	M146-[2.18]	M82-[2.40]	F238 [4.71]	M227 [4.36]	M120 [4.50]	M417 [3.24]	F/Q/M
^ #^D 133 *[2.58]	E81-[2.86]	P108-[2.49]	D106-[2.96]	E147 [5.39]	E-83-[2.83]	E239-[2.54]	E228-[2.88]	E121-[2.52]	E418-[3.05]	D/E-well conserved
^ #^Y 134 *[3.9]	F82 [5.08]	F109 [4.65]	L107 [3.03]	L148 [3.63]	L84 [4.73]	M240 [3.57]	Y229 [3.81]	Y122 [5.50]	Y419 [3.44]	F/L/M/Y
^ #^V 135 *[3.05]	C83-[3.19]	M110-[3.13]	M108-[3.03]	M149-[2.51]	L85-[2.97]	L241-[2.84]	A230-[3.20]	V123-[2.69]	V420-[2.64]	C/M/L/A/V
^ #^T 138 *[5.6]	D86-[2.74]	D113-[2.89]	D111-[2.77]	N152-[2.45]	S88-[2.91]	N244 [4.37]	E 234-[2.63]	E127-[3.01]	D424-[2.99]	D/N/S/E
^ #^R 141 *[3.10]	K89 [5.45]	K116 [5.32]	K 114 [3.92]	Q 155 [4.03]	D 91 [4.42]	D247 [5.51]	F237 [4.76]	S130 [5.50]	Y427 [>6]	R/K/Q/D/F/S/Y
^ #^Q 185 *[2.98]	Q130-[2.66]	G 154 [4.71]	S153-[2.71]	S193 [4.42]	D132 [3.84]	E291 [4.66]	E278-[3.11]	E170 [2.30]	D467-[3.23]	Q/G/S/D/E
^ #^N 186 *[3.45]	N131 [4.33]	N155 [>6]	N154 [3.98]	N194 [4.09]	N133 [2.88]	N292 [3.39]	N279 [5.38]	N171 [3.22]	N468 [4.22]	N-well conserved
^ #^L 188 *[5.8]	L133 [5.76]	A157 [5.79]	L156 [>6]	V196 [>6]	L135 [5.29]	L294 [>6]	M281 [5.54]	L173 [5.88]	M470 [4.33]	L/A/V/M
^ #^C 199 *[4.63]	A143 [5.93]	L167 [>6]	C166 [4.09]	L206 [5.20]	I148 [6.06]	V306 [2.70]	T291 [3.83]	T183 [3.62]	A480 [4.27]	C/A/L/I/V/T
^ #^D 200 *[4.84]	N144 [3.72]	D168 [5.04]	D167 [3.79]	D207 [4.19]	D149 [2.97]	D307-[2.72]	D292 [3.11]	D184 [3.96]	D481-[2.59]	D-well conserved

22	16	19	16	15	18	18	21	22	18	Number of interacting residues with ATP within 6 Å

^#^Indicates residue type and number; *indicates distances in Angstroms.

**Table 6:** Binding site residues of GSK3*β* interacting with 7 inhibitors when docked in the ATP Pocket.

Residues in the ATP binding site of GSK3*β*	ATP	Inhibitor 1	Inhibitor 2	Inhibitor 3	Inhibitor 4	Inhibitor 5	Inhibitor 6	Inhibitor 7	Number of interactions each residue has across inhibitors (within 6 Å)
I 62	4.4	—	3.62	2.35	4.09	3.98	2.90	2.84	6
G 63	5.4	—	—	—	—	—	4.50	3.20	3
N 64	5.24	—	—	—	—	—	—	4.56	4
G 65	4.03	—	—	—	—	—	—	—	1
S 66	2.82	—	—	—	—	—	—	—	1
F 67	3.21	—	—	—	—	—	—	—	1
V 70	5.9	4.09	—	5.20	—	—	5.37	—	4
A 83	5.46	4.96	5.51	—	5.80	—	5.03	—	5
K 85	2.64	3.03	—	3.21	5.02	—	—	2.84	5
E 97	4.59	—	—	—	—	—	—	—	1
V 110	5.1	—	3.35	—	—	—	—	—	2
L 132	5.7	—	4.41	5.69	—	—	5.11	—	4
D 133	2.58	2.62	2.88	3.21	2.87	—	3.03	—	6
Y 134	3.9	—	—	—	2.77	—	5.65	—	3
V 135	3.05	2.39	2.90	2.65	2.72	4.81	2.59	5.72	8
T 138	5.6	—	—	—	—	—	—	—	1
R 141	3.12	4.91	2.17	—	3.75	4.51	5.15	3.81	7
Q 185	2.98	—	—	2.99	5.89	—	2.94	2.90	5
N 186	3.45	—	—	4.64	—	—	4.368	—	3
L 188	5.8	4.03	5.12	—	—	—	—	—	3
C 199	4.63	4.05	—	—	—	—	—	—	2
D 200	4.84	2.70	4.57	3.45	—	—	—	—	4

Number of interacting residues across various inhibitors (within 6 Å)	22	9	9	9	8	3	11	7	—
